# Mechanisms of Lifespan Regulation by Calorie Restriction and Intermittent Fasting in Model Organisms

**DOI:** 10.3390/nu12041194

**Published:** 2020-04-24

**Authors:** Dae-Sung Hwangbo, Hye-Yeon Lee, Leen Suleiman Abozaid, Kyung-Jin Min

**Affiliations:** 1 Department of Biology, University of Louisville, Louisville, KY 40292, USA; leen.abozaid@louisville.edu; 2 Department of Biological Sciences, Inha University, Incheon 22212, Korea; 319127@inha.ac.kr

**Keywords:** aging, lifespan, longevity, calorie restriction, fasting

## Abstract

Genetic and pharmacological interventions have successfully extended healthspan and lifespan in animals, but their genetic interventions are not appropriate options for human applications and pharmacological intervention needs more solid clinical evidence. Consequently, dietary manipulations are the only practical and probable strategies to promote health and longevity in humans. Caloric restriction (CR), reduction of calorie intake to a level that does not compromise overall health, has been considered as being one of the most promising dietary interventions to extend lifespan in humans. Although it is straightforward, continuous reduction of calorie or food intake is not easy to practice in real lives of humans. Recently, fasting-related interventions such as intermittent fasting (IF) and time-restricted feeding (TRF) have emerged as alternatives of CR. Here, we review the history of CR and fasting-related strategies in animal models, discuss the molecular mechanisms underlying these interventions, and propose future directions that can fill the missing gaps in the current understanding of these dietary interventions. CR and fasting appear to extend lifespan by both partially overlapping common mechanisms such as the target of rapamycin (TOR) pathway and circadian clock, and distinct independent mechanisms that remain to be discovered. We propose that a systems approach combining global transcriptomic, metabolomic, and proteomic analyses followed by genetic perturbation studies targeting multiple candidate pathways will allow us to better understand how CR and fasting interact with each other to promote longevity.

## 1. Introduction

### 1.1. Opening Sentences

Almost all organisms, except for a few species including perennial plants, lobsters, quahog, rockfish, and Testudinidae, undergo a series of biological processes referred to as “aging” and “senescence.” [1]. Biological aging is generally defined as “a series phenomenon of functional, structural, and biochemical changes that occur throughout cells and organs, disrupting homeostasis in the body and ultimately leading to death” [2]. Prior to the early twentieth century, studies on human aging were not considered important because humans lived for a relatively short period of about 35 to 45 years. Since that time, technology and human medicine have greatly advanced, the human lifespan has increased, and research into human longevity and healthy living has increased. One of the breakthroughs of the research is that the aging process can be retarded by dietary manipulations.

### 1.2. History of Dietary Manipulations for Health and Longevity

In the early 1900s, there was some evidence that dietary manipulations affect health and longevity of organisms. Reduction of food intake decreased the occurrence of cancers in rodents [3], and increased the lifespan in aged female rats [4] and fruit flies [5]. The basic concept of caloric restriction (CR) was founded in the late 1930s. Ingle et al. reported that the reduction of food intake increased the lifespan of planktonic cladoceran, *Daphnia longispina* [6], and McCay et al. showed that restricted diet extended the lifespan of rats two fold compared to rats on a normal diet [7]. Since the late 1930s, the term CR has become more widely used, and, in the 1940s, many researchers reported that CR retarded or prevented the onset of age-related diseases such as kidney disease, tumors, and leukemia [8,9,10,11,12]. From the 1950s to the 1980s, the longevity effect of CR was also reported in other species. CR decreased the mortality rate in *Tokophrya infusionum* (Protozoan) [13], *Philodina acuticornis* (rotifera) [14], *Lebistes reticulates* (fish) [15], *Caenorhabditis elegans* (nematode) [16], *Rattus norvegicus* (rat) [17,18], and *Mus musculus* (mouse) [19,20]. In addition to limiting the feeding amount, controlling the feeding period (e.g., intermittent feeding) was also researched during these decades [18,20,21,22,23]. In the 1980s, several sources of evidence started to indicate that the dietary composition was the controlling determinant for the longevity effect of CR, and the term dietary restriction (DR) began to be widely used. Several studies have shown that reduced calorie intake by alteration of nutrient content, such as fat, carbohydrates, or amino acids, can have different effects on longevity in model animals [24,25,26]. In the 1990s, results of studies into the effects of CR in rhesus monkey (*Macaca mulatta*), non-human primates (NHP) were published by three groups—the National Institute on Aging (NIA) [27], the Wisconsin National Primate Research Center (WNPRC) [28], and the University of Maryland [29].

In the 2000s, the term intermittent feeding underwent a slight change and became intermittent fasting (IF). IF is a dietary manipulation that cycles between periods of ad libitum feeding and periods of fasting, including alternate-day fasting (ADF) and periodic fasting (PF) [30]. Although the effects of IF on health and longevity have not been elucidated as clearly as those of CR, there is evidence indicating a positive effect of IF on aging [31,32]. Recently, the concept of IF merged with that of the circadian rhythm and a new diet regimen, time restricted feeding (TRF), has emerged. TRF is a slight variation of IF interventions in which food intake is limited to 12 h each day without a change in the total calorie intake of the normal diet [31,32,33,34,35]. TRF has been reported to reduce the incidence of aging-related diseases and delay aging without an actual reduction in food intake.

### 1.3. Key Determinant of Lifespan Regulation through Diet Manipulation

CR regards the daily caloric intake per se as a key determinant in lifespan regulation. For example, a reduction of calorie intake without a reduction of protein intake increased the lifespan of rats [25], and lifespan was not altered in rats fed isocaloric diets in which either fat or mineral components had been reduced [26,36]. These studies indicated that the total calories are a key determinant in regulating the lifespan of rats. However, recent evidence had indicated that the amount of calorie intake might not be a key determinant of lifespan regulation by CR. The lifespans of rats and fruit flies have been increased by nutritional changes or protein reduction while providing the same calorie intake [37,38,39,40,41]. Moreover, the results of several studies have suggested that amino acids are key modulators of lifespan in organisms [42,43]. Furthermore, reducing only one type of amino acid, methionine, is sufficient enough to increase the lifespan of yeast, nematodes, fruit flies, and rodents [44,45,46,47]. Beneficial effects of TRF on health and longevity indicated that there might be a third determinant in lifespan extension, other than total calories or nutrient composition, since TRF exerts its effect without exhibiting notable changes in total calories or nutrient composition [31]. A more thorough investigation into the key determinant(s) of nutrient restriction effect is necessary. 

## 2. Animal Models and Protocols of Dietary Manipulation

### 2.1. Yeast (Saccharomyces Cerevisiae)

Yeast aging is classified into two different types as replicative and chronological aging [48]. Replicative aging is defined by the number of daughter cells produced by a mother cell, while chronological aging is defined by the time in which a nondividing cell can maintain viability. Although two yeast aging paradigms have been used in aging studies, replicative aging is more widely used in CR-related aging studies. Generally, CR in yeast is performed by reducing the glucose level in growth medium, which commonly contains 2% peptone, 1% yeast extract and 2% glucose. The concentrations of glucose are reduced to ~0.5–0.005% for CR [49]. In these settings, replicative lifespan of budding yeast was extended by about 10 times in the low-dose glucose medium compared to the lifespan of control [50,51,52,53]. Yeast is also cultured in water in order to undergo fasting [54].

### 2.2. Nematode (Caenorhabditis Elegans)

*C. elegans* has several advantages in aging studies—a relatively short lifespan/reproductive cycle, a translucent body, it is easy to culture, has a small genome, and there are many available mutants [55]. DR is mainly performed in nematodes by controlling the concentration of the bacteria such as *Escherichia coli* in the media that they feed [54,56]. In the worms, genetic perturbations that mimic DR were also introduced by inhibiting specific nutrient transporters [57] and reducing pharyngeal pumping [58]. For IF, worms are placed every other day in medium with and without bacteria [59,60]. This IF regimen (alternate 2 days eating/ 2 days fasting) successfully extended lifespan in the worms [59,60]. Furthermore, chronic fasting also increased the lifespan of worms compared to normal diet-fed worms [61,62].

### 2.3. Fruit Fly (Drosophila Melanogaster)

The fruit fly, *D. melanogaster,* is another invertebrate model organism widely used for aging and dietary intervention studies [63]. Similar to *C. elegans*, the fruit fly also has many advantages such as a relatively short lifespan and high productivity. However, compared to *C. elegans*, the fruit fly has more complicated and diverse tissues such as the heart and kidney that are functionally homologous to mammals [63]. Gene manipulation and editing tools are also readily available to study the genes of interest in a time- and tissue-controlled manner [63]. Furthermore, their simple food composition allows for easy manipulation of the food component in experiments. Although the composition of the food medium is diverse among laboratories, the most general method for DR supplementation in the fruit fly is dilution of the food ingredients including yeast as a protein source, sugar, or fat from an ad libitum medium. Food reduction or diluted food has also been consistently shown to extend the lifespan in fruit flies [40,64,65]. Furthermore, limiting amino acids such as methionine or limiting protein sources were sufficient to increase the lifespan of fruit flies [40,41,46,66]. A relatively diverse fasting study design can be carried out in fruit flies. In the case of ADF, food is provided every two days and fasting is performed for 24 h. Recent studies have found that a 2-day fed:5-day fasted IF regime [67] and a TRF regime with daily access to food during the day and water access during the night [68] can be implemented in fruit flies. In the IF regime’s case, flies were treated for IF for the first 30 days of adulthood and then switched to an ad libitum diet due to high mortality by fasting in older flies [67]. In this regimen, IF increased the lifespan of fruit flies [67]. However, a 3 h or 6 h starvation during the day was not enough to extend the lifespan [65]. Additionally, TRF did not increase the median lifespan of fruit flies, although TRF improved the muscle performance and attenuated age-related cardiac dysfunction [31,68].

### 2.4. Rodents

Although research results showing longevity manipulation by dietary modulation in nematodes and fruit flies are thought-provoking and motivating, the complexities of human physiology block the direct application of such results in humans. In this regard, rodents can fill some of the gaps between them and humans because, compared to fruit flies, nematodes, and yeast, rodents have a closer phylogenic relationship to humans and greater similarities in their physiological features and process. Many studies have shown beneficial effects of CR/DR on aging in rodents. For example, CR/DR reduced the incidence of age-related diseases such as cancer, neurodegenerative diseases, and cardiovascular diseases and prolonged lifespan by 30% in rats and 15% in mice [24,69,70,71]. Rodents, including mice and rats, were the first experimental model systems used to investigate the effect of CR on lifespan [7]. Generally, to conduct CR in rodents, the total consumed volume of food is thoroughly controlled so that 20–50% of calories are reduced compared to ad libitum food administration. [72,73]. In addition to this traditional CR administration, trials modulating macromolecule composition such as proteins or carbohydrates were also attempted. Similarly, reducing the concentration of specific amino acids such as methionine or tryptophan is another form of dietary modulation and was shown to extend lifespan [42,47,71,74,75,76,77,78]. To assess the effects of fasting regimen in rodents, IF can be conducted so that rodents are provided with only water or minimal nutrients for less than 24 h followed by a normal diet period of 48 h, whereas PF can be conducted so that rodents are fasted for approximately 48 h, returned to normal feeding and then fasted again at least one week later [79]. To conduct TRF, food access can be regulated by transferring mice daily between cages with ad libitum food and cages with water only [80,81]. In rodent models, the effects of IF on lifespan are not yet conclusive. IF with every other day fasting or fasting for one day every three to four days extended the lifespan of rodents [82,83,84,85]. However, a study showed that IF introduced at 10 months of age had no effect on mean lifespan in C57BL/6J mice or decreased the lifespan in A/J mice [83]. Unlike IF, multiple studies showed that TRF inhibits several chronic diseases and tumor progression and increases lifespan in rodents [86,87,88].

### 2.5. Non-Human Primates

The use of NHP in dietary studies provides unique evidence that cannot be obtained by studying a lower-order model animal. Although the results of NHP studies have high reliability in human applications, NHP studies can encounter several technical, financial and ethical difficulties. Three independent groups, the NIA, the WNPRC, and the University of Maryland have investigated, or are currently investigating, the beneficial effects of CR on NHP by using the rhesus monkey model. A research group at the University of Maryland have focused on the effects of short-term CR on obesity and diabetes [89,90], while the NIA and WNPRC have been investigating the effects of CR in rhesus monkeys throughout their entire lifetime. Although the rhesus monkeys in the CR groups were provided with about 70% food compared to ad libitum groups in both the NIA and WNPRC studies, there is a key difference between them in terms of dietary composition [91,92,93]. The NIA provided unpurified natural ingredient-based food, while the WNPRC provided a purified diet to monkeys [91,92,93]. Although the exact information of food ingredients is not available in natural ingredient-based food, it provides phytochemicals and minerals which might have beneficial effects on health and lifespan. On the other hand, a purified diet has an advantage in that nutrient composition of the diet is more defined, allowing the manipulation of specific components of the diet. In addition, the NIA provided approximate ad libitum intake considering their age and bodyweight for the maturing control monkeys without overfeeding, but the WNPRC established the ad libitum reference for each individual and implemented CR based on individual standards [91,92,93]. Lifelong CR in rhesus monkeys led to lifespan extension at the WNPRC [91], but there was no lifespan extension effect by CR at NIA [92]. The NIA used the food that was lower in calories and fat, and higher in protein and fiber compared to food used by the WNPRC. These dietary manipulations conducted at the NIA led to a longer lifespan of the control old-onset groups from the median lifespan of rhesus monkey. The median lifespan of rhesus monkey was similar to what was previously reported as the 90th percentile of this species (~35 years old). In addition, juvenile/adult males without CR in the NIA showed similar median lifespans compared to the lifespan of monkey with CR in the WNPRC. Thus, it suggests that the difference in diet between the control and the CR group was insufficient to change lifespan. However, since the NIA uses rhesus macaques of various ages, sex, and different genetic backgrounds (Indian and Chinese), it showed results that can compare the effect of CR according to the differences in age/sex/genetic background. Although the results of the effect of CR on the lifespan of rhesus monkey were different, both groups present health benefits of CR such as loss of weight and fat, and reduced risk of cancer and cardiovascular disorder. Thus, if all variables were controlled, it was suggested that CR can robustly increase lifespan in monkeys and also suggest applications in humans [93].

## 3. Dietary Manipulations for Human Application

Many studies have shown that dietary manipulation can retard the aging process through some well conserved mechanisms in diverse organisms from yeast to NHP. The determination of conserved mechanisms that produce beneficial effects of dietary manipulation in humans would require additional investigation, due to the limited number of studies examining the effects of CR/IF in humans. However, several epidemiological and cross-sectional studies using centenarians and individuals who volunteered CR practice indicate the beneficial effect of CR in humans. Epidemiological data can be gathered from people who follow food restrictions due to religious guidelines. For example, Muslims ingest no food or water for approximately 15 h between sunrise and sunset for a month during Ramadan every year. Thus, this long-term food restriction during Ramadan could be considered a human IF model.

Some studies have shown that Ramadan fasting has the effect of promoting human health [94]. A Comprehensive Assessment of the Long-term Effects of Reducing Intake of Energy (CALERIE) research program was designed to systematically investigate sustained CR effects in healthy volunteer humans over a two-year period [95]. The CALERIE program produced several results that demonstrate the beneficial effect of CR on aging and health in humans, including observation of an increase in metabolism and a decrease in oxidative stress [96,97]; however, the study did not indicate the presence of beneficial effects of CR on age-related bone and muscle impairment [98,99]. Additionally, some studies have shown that IF can improve metabolic health and physiological function in humans. IF reduced fat mass, lean mass, and body weight in healthy humans and obese patients [33,100,101,102,103,104]. Similarly, IF improved lipid and glucose metabolism, reduced inflammatory response, lowered blood pressure, and improved cardiovascular health [102,105,106,107,108,109]. Several studies have shown that IF is an effective intervention, especially for people who are overweight or diabetic. IF reduced overall fat mass and decreased insulin resistance [103,110,111,112,113]. Some researchers also conducted the studies to evaluate the effects of TRF on human health, and demonstrated that TRF improved insulin sensitivity, blood pressure, oxidative stress, and quality of life in overweight or diabetic adults [35,114,115]. Results of the studies also showed that TRF improved cardiovascular function and other indicators of healthspan (e.g., walking distance and heart rate) in healthy middle-aged and older adults [116] although weight loss observed with other IF methods were not accompanied by TRF. These results suggest that IF including TRF may be a promising manipulation to extend the healthspan of humans.

## 4. Molecular Mechanisms of CR and IF

The ultimate goal for animal studies on CR/IF is to identify the conserved molecular mechanisms that can extend the healthspan of humans. Healthspan, the period of life that is free from disease, is measured by examining declines of functional health parameters and disease states. Because healthspan is a multifactorial complex phenotype that is significantly affected by genotypes (G) and environmental factors (E) as well as complicated interactions between them (G × E), measuring healthspan often gets complicated [117]. Furthermore, delayed functional aging in one parameter is not always necessarily linked to the extension of healthspan in different health parameters [117]. In fact, by depending on the types of health parameters and experimental approaches, different healthspan results were observed from the studies that used the same long-lived mutant animals [117]. Unlike healthspan, lifespan is unequivocally recorded by simply following the mortality of individual organisms. Lifespan extension in animal models is strongly correlated with a decrease in morbidity and an increase in health. Therefore, although we believe that results of health-related parameters from animal CR/IF studies are likely to be translatable to human healthspan, we will focus on the mechanisms of lifespan extension in animal models in this manuscript.

**Figure 1:**
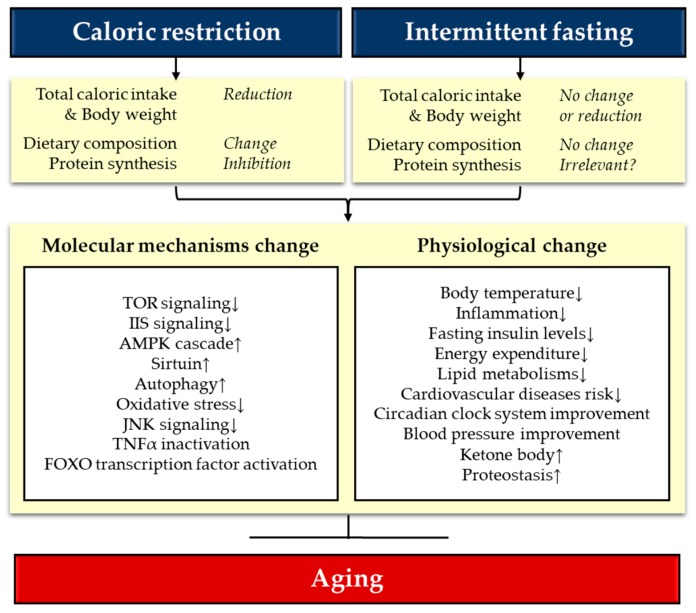
Possible anti-aging mechanisms of caloric restriction (CR) and intermittent fasting (IF). Different dietary interventions by CR and IF result in similar molecular and physiological changes that promote longevity in model organisms. Patterns of individual dietary, metabolic, molecular, and physiological parameters can be different depending on the types of CR and IF as well as the animal models. See the main text for details.

Although not complete, studies for the last two decades on CR have provided a great amount of details about the mechanisms of CR. Recent advances in OMICs and bioinformatic techniques followed by organism level genetic perturbation analyses significantly extended our knowledge on the molecular mechanisms that mediate lifespan extension by CR. A current understanding is that CR works through the key nutrient and stress-responsive metabolic signaling pathways including IIS/FOXO, TOR, AMPK, Sirtuins, NRF2, and autophagy. While these pathways regulate CR independently, cross-talks among these pathways as well as upstream master networks such as circadian clock were also suggested to regulate lifespan extension by CR. Although the number of reports on IF is less than CR, recent studies clearly demonstrated that IF also extends lifespan in both vertebrate and invertebrate model organisms [60,67,79,83,118,119]. Notably, increased survival by nutrient deprivation was also observed in prokaryotic *E.coli* cells, emphasizing that fasting-related lifespan extension is evolutionarily conserved [79]. However, there is still a lack of comprehensive understanding for the mechanisms responsible for lifespan extension by IF. As nutrient-dependent interventions, CR and IF were suggested to share a common strategy: the reduction of caloric intake and nutrients that limit longevity. In fact, CR and IF also result in common metabolic and physiological changes in multiple tissues and organs (Figure 1) [32]. For example, ketone bodies, insulin sensitivity, and adiponectin are increased while insulin, IGF-1, and leptin are decreased. Overall inflammatory response and oxidative stress are reduced by both regimens [32]. They also cause similar behavioral changes such as increased hunger response and cognitive response [32]. Accordingly, it is widely accepted that common molecular mechanisms may mediate the lifespan extension by CR and IF. A proposed model for the mechanisms underlying the lifespan extension by CR and IF relatively follow the notion that both CR and IF alter the activity of common key metabolic pathways, namely, TOR, IIS, and sirtuin pathways (Figure 1) [120]. However, there must be independent mechanisms as well due to one major difference between CR and IF in that IF aims to extend lifespan without an overall reduction in caloric intake by taking advantage of the molecular pathways that respond to fasting [30,32,121].

Chronic CR that results in the extension of healthspan and lifespan usually involves a body weight loss in animal models [119]. Body weight loss is also often observed in animals under IF [119]. This is an important issue in both practical and mechanistic perspectives. Although a modest body weight loss may be beneficial for overall health, a severe loss of body weight may counteract beneficial effects on other health parameters. Mechanistically, it is possible that CR and IF result in extension of healthspan and lifespan at the cost of body weight reduction. In this sense, it is interesting to note that a loss of body weight can be decoupled from other beneficial effects by IF [30,114]. This raises an important question of whether fasting by itself may induce some, if not all, extension of healthspan and lifespan at least by IF. Although a weight loss was observed in the participants of the CALERIE trial (also seen in Section 3), the weight loss was mild and within the normal range of health while improving other health parameters [95,96,97]. Therefore, although further investigations are required for the reciprocal relationship between body weight and the efficacy of CR/IF, we favor the idea that that body weight reduction by CR and IF are side effects that are not the mechanistic determinant for the benefits of CR and IF.

CR and IF significantly reorganize genomic, metabolomic, and proteomic landscapes in local tissues as well as in the global organism level in an age, sex, and strain-dependent manner. However, these molecular changes in gene expression, metabolites, and proteomes do not necessarily represent whether those changes are causal factors for CR- and IF-mediated lifespan extension. Genetic perturbation studies in animal models must be followed in order to link them to lifespan regulation by CR and IF. Therefore, in this review, we will primarily focus on the molecular pathways that were genetically tested for CR and IF effects on lifespan, leaving out much of correlative studies describing the physiological and metabolic traits affected by CR and IF. Because genetic perturbation studies and OMICs data for IF are significantly less than those of CR, we will first discuss molecular mechanisms of CR followed by whether those mechanisms overlap with IF.

### 4.1. AMPK-TOR Signaling

In eukaryotes, the target of the Rapamycin (TOR) pathway plays a central role in nutrient and energy sensing to control cellular and organismal growth [122,123,124]. The TOR pathway regulates growth and metabolism by promoting protein synthesis in response to nutritional availability including dietary amino acids [124]. A number of genetic studies showed that suppression or downregulation of the TOR pathway extend lifespan in multiple model organisms including the yeast *S. cerevisiae* [54,125,126,127,128,129], the worm *C. elegans* [60,130,131,132,133,134,135,136,137,138,139,140,141,142], the fly *D. melanogaster* [143,144], and the mouse *M. musculus* [145,146,147,148]. As CR downregulates the TOR signaling cascade, it has long been suggested that CR may extend lifespan by at least partially suppressing the TOR pathway at the cost of reduced growth. In fact, mutant animals for the components of the TOR pathway were often shown to fail or decrease in lifespan extension by CR [54,125,126,127,128,129,136,141,143,144], indicating that the TOR pathway antagonizes the full benefit of CR-mediated lifespan extension. As a key amino acid sensing pathway, this may explain that restriction of protein alone, specifically by single amino acids methionine and tryptophan in the diet, were sufficient to extend lifespan.

In addition to amino acids, the TOR pathway is also regulated by cell energy status through AMP-dependent protein kinase (AMPK), a conserved energy sensor in eukaryotes [149,150]. Increased AMP:ATP ratio by energy depletion such as CR activates AMPK, which in turn inhibits the TOR pathway [149]. Thus, CR activates AMPK while suppressing the TOR cascade subsequently. Unlike the TOR pathway where it extends lifespan when suppressed, AMPK extends lifespan in model organisms when activated [136,151,152,153]. Importantly, similar to the TOR pathway, genetic perturbation studies also showed that AMPK mediates lifespan extension by CR. For example, lifespan extension by CR in worms was suppressed in the mutant worms for *aak-2*, one of the catalytic subunits of AMPK [136]. However, it is interesting to note that another type of CR in worms (i.e., feeding diluted bacteria in liquid culture) did not require AMPK signaling to extend lifespan [154]. Although this discrepancy needs further investigation particularly into their methods including the nutritional value in each type of the CR protocols, it is possible that non-overlapping mechanisms between CR and IF may be responsible. In other words, fasting-related mechanism independent of CR may contribute to this difference. In this sense, it is interesting to note that mild nutritional stress through feeding 2-deoxy-D-glucose (2-DG) or food deprivation, which mimic fasting, extended lifespan in worms through AMPK signaling [155,156]. An indication for this explanation can be drawn from mammalian studies. While acute starvation readily activates AMPK, activation of AMPK depends on the duration and type of CR [157]. In some cases, extended CR failed to activate AMPK [157]. Thus, it is possible that the AMPK-TOR dependent lifespan extension could partially be due to the mechanisms induced by fasting, parts of which may be independent of CR. Supporting this hypothesis, it is noteworthy that Honjoh et al. showed that lifespan extension by IF (by every-other-day feeding) was dependent on RHEB, a small GTPase protein that activates the TOR pathway by directing binding to the TOR Kinase [158], at least in worms [60]. As they also showed that RHEB-dependent IF-mediated lifespan extension was partially due to IIS/FOXO signaling, their results support the idea that tightly regulated networks between IIS/FOXO and TOR signaling cascade may mediate both DR and IF-dependent lifespan extension.

### 4.2. IIS-FOXO Signaling

In mammals, growth hormone (GH) secreted from the pituitary gland promotes somatic growth by activating a cascade of downstream hormonal signaling such as Insulin/Insulin-like growth factor-1 signaling (IIS) [120,159]. Activated IIS signaling cascade by GH mediates the translocation of its main downstream targets, forkhead box protein O (FOXO) transcription factors, to the cytoplasm from the nucleus [160]. In the absence or reduction of GH/IIS signals, the FOXO transcription factors translocate into the nucleus and promote the expression of their target genes involved in cell death, cell cycle arrest, DNA repair, stress resistance, and detoxification [160], all of which are attributed to promote longevity by switching organismal metabolic status from somatic growth to maintenance [161]. Although there is no system equivalent to GH in lower organisms such as yeast, worms, and flies [120], a number of observations reported for the last two decades strongly support the idea that downregulation of IIS and activation of FOXO transcription factors extend lifespan in these animal models (reviewed in [120,159,162,163]). In fact, of the > 40 genetic mutations that have been reported to extend lifespan in the mouse and the rat models, approximately one third of them are involved in GH and IIS [164]. Because CR reduces GH and IIS [164], it is generally accepted that CR extends lifespan by limiting GH/IIS signaling and subsequently expressing pro-longevity genes by activating FOXO transcription factors [165]. To date, there are mixed results reported for the question of whether the IIS-FOXO signaling cascade is responsible for CR-mediated lifespan extension. For example, Bonkowski et al. reported that dwarf mice with targeted disruption of the GH receptor failed to extend overall, median, or average lifespan by CR (food reduction by 30% compared to ad libitum) [69], suggesting that CR extends lifespan by downregulation of IIS. Alternatively, in another study, CR (30% CR) further extended the lifespan of the long-lived dwarf mice with GH production that was selectively suppressed in the pituitary gland, spleen, and thymus [166], suggesting that lifespan extension by GH suppression may occur through an independent mechanism of CR. Alternatively, these results also imply that GH signaling in other tissues such as the liver and testis should be also suppressed for a full benefit of lifespan extension by CR [166], raising an important question regarding the tissues critical for CR-mediated lifespan extension. Interestingly, these data show a clear dissociation of lifespan extension by GH suppression from its dwarfism (small body size caused by GH suppression), opening an important possibility that CR may extend lifespan without the cost of growth reduction. Similar to the dwarf mice mutant for the GH receptor [69], CR failed to extend lifespan of both heterozygous and homozygous mutant mice for FOXO3 [167], showing that IIS-FOXO signaling is indeed required for the full benefit of CR-mediated lifespan extension. More complicated observations were reported in lower organisms. In flies, multiple studies suggest that although IIS-FOXO signaling modulates longevity response to CR, it appears not to be the main player of CR [168,169,170]. In worms, it is still inconclusive whether IIS-FOXO is required for CR-mediated lifespan extension because mutant worms for DAF-16, the sole ortholog of FOXO transcription factors, showed a different longevity response depending on the types of CR [154]. While a relatively considerable amount of research has been done on the relationship between IIS-FOXO and CR-dependent lifespan extension, no direct genetic studies testing whether IIS-FOXO mediates IF-dependent lifespan have been reported. However, functional studies characterizing the reciprocal effect between IF and IIS-FOXO signaling suggests that IIS-FOXO may be at least partially responsible for IF-dependent lifespan extension. For example, in mammals, key metabolic and physiological changes attributed to lifespan extension by CR include increased insulin sensitivity, stress resistance, and immune function with reduced inflammation. Recent studies demonstrated that IF also shows these beneficial changes, displaying a promising prospect that IF may also increase lifespan through IIS-FOXO signaling.

### 4.3. Sirtuins

Sirtuins, silent information regulator 2 (sir2) proteins, are protein deacetylases that require NAD^+^ as a cofactor for the deacetylation reaction [171]. Because NAD^+^ and its reduced form NADH are involved in many important cellular metabolic pathways, sirtuins function as metabolic sensors that represent the metabolic state of the cell. As NAD^+^ accumulates under nutritional stress and activates sirtuins [172], it was suggested that activation of sirtuins may extend lifespan, possibly through the mechanisms that extend lifespan by CR and/or IF. In fact, it was shown that genetic overexpression of sirtuins extended lifespan in multiple model organisms including yeast [173], worms [50,174,175,176,177,178,179,180,181,182], flies [178,183,184,185], and mice [186,187]. Similarly, pharmacological activation of sirtuins by feeding resveratrol extended lifespan in some of these animals [178,188]. Furthermore, it was also shown that the sirtuin family genes were required for the lifespan extension by CR in these animal models [50,178,183,184,185]. For example, when SIR2 was deleted, CR by glucose dilution failed to extend lifespan in yeast [50]. However, it is interesting to note that, while a milder CR (0.5% glucose) in yeast required SIR2 for lifespan extension [50], a severe form (0.05% glucose) of CR extended lifespan independent of SIR2 [189]. It would be important to test whether this severe form of CR extend lifespan by the mechanisms related to fasting. In this case, it would also be critical to identify the threshold concentration of glucose that differentiates fasting from CR. Characterizing global changes in transcriptome and metabolome between these *sir2*-dependent mild CR and *sir2*-independent severe CR (aka fasting) would be also critical to better understand the relationship between CR and fasting. In flies, increased lifespan by *sir2* overexpression was not further extended by CR [183]. On the other hand, CR failed to extend the lifespan of null mutant flies for sir2 [183]. It was also shown that genetic knockdown of *sir2* in fat body suppressed the lifespan extension by CR [185]. These reports support the idea that *sir2* plays a critical role in CR-dependent lifespan extension. In worms, whether *sir-2.1* (the ortholog of *sir2* in yeast and flies) is necessary for CR-mediated lifespan extension or not was dependent on the type of CR-treatment [154]. It would be interesting to test whether the type of CR that does not require *sir-2.1* extends lifespan by activating the pathway that extends lifespan by fasting. Despite all of these observations that support the idea that sirtuins are important mediators of CR, there are conflicting claims about the role of sirtuins in pro-longevity and CR-mediated lifespan extension in lower eukaryotic organisms [189,190,191]. This discrepancy may be due to differences in dosage of sirtuins, tissue septicity, and CR administration protocols [189,190,191,192]. For example, lifespan extension by overexpression of sirtuins depends on the levels of sirtuins [184,185,192,193]. When *sir2* was expressed over 45 fold, it resulted in a shortened lifespan while a modest overexpression up to 11 fold increased lifespan [193]. Therefore, the impact of sirtuins on aging, CR-mediated, and possibly IF-mediated lifespan extension needs to be thoroughly studied [189,190,191,192]. In mice, knockout mutants for SIRT1, one of the seven mammalian sirtuins homologous to invertebrate sirtuins [194], failed to extend lifespan under CR [195,196], confirming that sirtuins’ role in CR-mediated lifespan extension is conserved across species. In addition, similar to the lower organisms, multiple studies demonstrated that activation of sirtuins extended lifespan in mice [186,187]. Overall, if some degree of variability in published data is tolerated [189,190,191], it can be concluded that the sirtuin pathway is key for CR-mediated lifespan extension in both invertebrate and vertebrate model organisms. However, despite the observations that NAD^+^ levels are increased by fasting and that sirtuins are involved in the benefits of fasting in physiological and pathological level [32,197], whether SIRT1 or the other mammalian sirtuins (SIRT1-6) play a role in IF-mediated lifespan extension is poorly understood. There is no lifespan data yet shown in animal models that specifically tested for the involvement of sirtuins in IF-mediated lifespan extension. It was recently revealed that fasting induced *dSirt4* (a *Drosophila* sirtuin family member localized to mitochondria) and over-expression of *dSirt4* extended lifespan [198]. It would be of great interest to test whether *dSirt4* mediates the CR- and IF-dependent lifespan extensions. Furthermore, considering the fact that the levels of sirtuins can result in opposite results in lifespan [193], it would also be important to profile the expression levels of sirtuins by different types of CR and IF.

### 4.4. Circadian Clock

Circadian (~24 h) clocks control a wide range of rhythmic metabolic, physiological, and behavioral parameters by communicating timing information via rhythmic transcription of output genes [199]. The misalignment of these internal clocks with 24 h environmental cycles are known to adversely impact metabolism, aging, and age-related disease [200,201]. Because the circadian clock orchestrates daily metabolism in response to cellular needs and nutritional availability, it was proposed to mediate the beneficial effect of CR [191,202]. A series of recent observations suggested that the circadian clock may play a master role in CR-dependent lifespan extension [203,204]. For example, it was shown that CR for two months in early life was sufficient enough to increase the amplitude of core clocks in the mouse liver [204,205]. As loss of rhythmic expression of clock-controlled genes (CCGs) is implicated as a cause of aging, these results suggest that CR may promote longevity by strengthening the rhythmic regulation of metabolism and physiology. In this regard, it is remarkable that CR failed to extend lifespan of knockout mice for Bmal1, one of the core circadian clock transcription factors [206], indicating that a functional circadian clock system is indeed necessary for CR-dependent lifespan extension in mice. Similar to mice, in flies, Katewa et al. reported that CR also increased the amplitude of core clock genes [203]. They also showed that genetic perturbation that increases clock function also resulted in lifespan extension in a diet-dependent manner [203]. Furthermore, they showed that homozygous mutants for *timeless*, a core clock gene in flies, failed to extend lifespan under CR to the level of wild type [203], indicating that circadian clock is also determinant of CR-dependent lifespan extension in flies. However, whether circadian clock is required for CR-mediated lifespan in flies needs cautious analysis as inconsistent results were reported, possibly due to uncontrolled environmental factors such as intestinal microbiome among the fly population [203,207,208]. With these observations in mice and flies, one important question is how exactly the circadian clock mediates the beneficial effect of CR. It is noticeable that transcriptional and post-transcriptional regulation of most known CR effectors such as GH/IGF-1, FOXO, TOR, AMPK, sirtuins, and NRF2 are directly or indirectly under the control of the circadian clock [32,202]. This raises the possibility for the circadian clock to play a master role in CR-mediated lifespan extension by simultaneously controlling these CR pathways. For example, in mice, cellular production of NAD^+^, a key co-factor of sirtuins that promotes CR-dependent lifespan extension, is under the circadian clock. During fasting at night, the NAD^+^ level is increased, which, in turn, activates sirtuins [32]. Similarly, nutritional input from feeding during the day increases ATP:AMP ratio and amino acid availability, thereby increasing the IIS and TOR pathways while suppressing the AMPK cascade. This process facilitates anabolic reactions and may promote aging. On the other hand, metabolism is switched to catabolic reactions by decreased ATP:AMP ratio and amino acid availability during fasting at night. Consequently, fasting at night suppresses the IIS and TOR pathways while activating the AMPK cascade and FOXO transcription factors, which subsequently give rise to anti-aging effects. Therefore, the circadian clock system may promote longevity by relaying the anti-aging signals induced by CR and IF.

One outstanding question is whether it is the total caloric/diet intake, rhythmic oscillation between feeding and fasting, or fasting itself (time and duration of fasting) that determines the beneficial effect of CR and IF. At least in mice, recent studies provided evidence that supports fasting as the key factor for CR- and IF-mediated lifespan extension. A systemic monitoring of food consumption behavior revealed that mice given the CR diet tended to limit their feeding time to a narrow temporal window, self-imposing and mimicking TRF [209]. Thus, mice under CR experienced a longer fasting time than when under AL diet [209], suggesting the possibility that it was not the calorie but the timing of food consumption or duration of fasting that confers longer lifespan in CR. Another study unequivocally demonstrated that mice under TRF extended lifespan even when they were under AL diet [88]. This study proved that controlling time-of-feeding can override the anti-longevity effect of caloric intake and is sufficient for lifespan extension [88]. This may explain why lifespan was not extended in mice when they were allowed to eat a hypo-caloric diet all day, although their overall caloric intake was comparable to that of CR [42]. Because these studies show that eating pattern (i.e., circadian fasting time and duration) rather than nutritional value (i.e., calorie and composition) determines lifespan, lifespan extension by CR and IF could occur at least partially through non-overlapping independent molecular mechanisms. Therefore, these observations strongly argue that molecular mechanisms responsible for lifespan extension by CR utilize some of the metabolic changes that occur during fasting. In this sense, lifespan extension by restricting specific nutrients such as methionine may also be due to changes in eating patterns that mimic TRF and IF as in Mitchell et al. [88]. With the evidence that restriction of caloric intake as well as specific nutrients such as methionine are sufficient to extend lifespan, these studies also indicate that there are both common and independent mechanisms underlying CR- and IF- mediated lifespan extension. Unlike CR studies in mice, where they have to fast once they consumed all the food that is given to them, CR in invertebrate models such as flies and worms allows them to have constant access to food. In fact, although there are daily rhythms in feeding behavior, flies do feed continuously over 24 h [210,211], removing the possibility that CR-mediated lifespan extension in flies is through the mechanisms by which IF extends lifespan. Furthermore, a genome-wide expression analysis revealed that global expression changes by CR and TRF differ from each other [212]. Importantly, this study also showed that the gene expression signature of TRF is also different from an extended starvation, raising the possibility that the molecular changes responsible for IF-mediated lifespan extension are different from that of CR, but also may not be from extremely severe fasting conditions. Gill et al. also reported that TRF ameliorates age-dependent heart failure by a mechanism independent of starvation and CR [212]. They showed that global transcriptional response to TRF is very different from that of starvation and CR [212]. Instead, they discovered that the circadian clock and clock-controlled TCP-1 ring complex chaperonin mediate the TRF effect. It will be of great interest to test whether TRF promotes longevity in flies, in which case these pathways might also mediate lifespan extension by TRF. Discovering the contribution of circadian clock to the benefits of TRF in Gill et al.’s study is not unexpected, considering the role of the circadian clock system to regulate daily metabolism and physiology in response to rhythmic environmental signals including the light:dark cycle and food consumption. Despite all of this compelling evidence, contribution of circadian clock to CR in worms and yeast is less understood due to their lack of a homologous system of a circadian clock pathway. However, they contain oscillatory metabolic fluctuations and behavior which need to undergo further studies for whether their CR response can be also modified by a circadian oscillatory mechanism [213,214,215].

## 5. Conclusions and Future Directions

### 5.1. Coordinated Regulation between IIS, TOR, AMPK, Sirtuins, and Circadian Clock

The ultimate goal of animal studies for CR and IF is to uncover evolutionarily conserved molecular mechanisms for the beneficial effect of CR and IF, and to eventually apply them to humans. Despite recent progress in our understanding of CR and IF, there are multiple challenges to overcome in order to achieve this goal. One such challenge is that there still lacks a comprehensive understanding of coordinated regulation among the key molecular pathways known and suggested to mediate CR and IF, namely, IIS, FOXO, TOR, AMPK, sirtuins, and the circadian clock. Molecular characterization of these pathways showed that they are tightly linked to and intertwined with each other in response to cellular nutritional state. However, the majority of animal studies performed so far on these pathways for the impact of CR and IF have been limited to testing and identifying single genes and pathways. Considering the impact of these pathways on systemic metabolism and physiology in many different tissues and organs, it is unlikely that a single gene or pathway is solely responsible for the lifespan extension by CR and IF. One way to solve this issue is to target multiple genes and pathways simultaneously [154,216]. For example, Hou et al. postulated that perturbation of multiple pathways would result in an additive or synergic effect in lifespan extension compared to the lifespan extensions by any single gene perturbation [217]. Using *C. elegans* as a model organism, they took advantage of the temporally resolved global transcriptome analysis followed by a systems biology approach. From this approach, they discovered that a combination of downregulation of IIS, downregulation of TOR, and upregulation of AMPK strongly resembled the transcriptomic change induced by CR [217]. Further genetic testing confirmed that lifespan was maximized when all of these perturbations were combined. More importantly, they also discovered that CR failed to further extend lifespan in these animals [42], showing that a simultaneous targeting of multiple candidate pathways may increase the power to detect hidden mechanisms for CR and IF.

### 5.2. Limits of Animal Studies for CR and IF

The amount of food that animals consume (meal size) and the time/duration of food consumption (meal timing) that animals take are key factors to interpret CR and IF results in animal models. Unlike rodent models where food is readily provided and removed from experimental animals, these parameters (i.e., meal size and meal timing/duration) are hardly controlled in the lower organisms widely used for CR and IF studies such as yeast, worms, and flies. Regardless of the method of choice for CR and IF, these animals basically feed ad libitum when they are provided food. A bigger challenge is that it is not practically easy to measure the amount of food they consumed, which is an important confounding factor to interpreting CR and IF data. An unignorable number of different, often contradictory, results from different strains and/or laboratory on CR and IF may be at least partially due to these factors. Importantly, these limits also put roadblocks on the translation of animal studies for CR and IF into human applications. In addition to these practical limits, the interspecies differences in physiology, metabolism, reproduction, and behavior between model organisms and humans serve as additional confounding factors for human translatability. For example, rodents have much higher metabolic rates than humans [218], yet similar fasting and feeding protocols are often used for IF. In addition to these intrinsic differences between model organisms and humans, intraspecies variations (differences in the population of the same species; also seen Section 5.3) often add to the complexity of human translation of animal studies. In flies, although some beneficial effects were observed by TRF (12 h of fasting during the dark phase of the day) on cardiac function and other metabolic and behavioral parameters such as body weight and sleep [212], an increased mortality was observed by 12 h of fasting in some young (<2 weeks) wild types flies (D.S. Hwangbo, unpublished data). On the other hand, some other wild type flies were strongly resistant to an extended period of fasting (up to 5 days), at least when they were young, during the IF regime of 2 day feeding:5 day fasting [67]. We speculate that, due to the confounding factors arising from the interspecies and intraspecies differences, the degree of beneficial effect of CR and IF on healthspan and lifespan in humans might not be equivalent to that of animal models [4,219]. Therefore, for the best working CR and IF protocols for human translations, we propose that multifactorial models should be developed to accommodate these confounding factors that interfere with the interpretation of animal results to human applications.

### 5.3. Individual Variations

From a practical perspective, IF is often thought of as a milder form of CR and generally considered to be easier for human implication. Beyond the evolutionary difference in metabolism and physiology between animals and human, potential interactions between genetic variations among human populations and the candidate mechanisms for CR and IF should not be overlooked. Human lifespan is affected by multiple genetic and non-genetic factors including population origin and interactions between the nuclear/mitochondrial genome and microbiomes [220]. It was suggested that only about 10–25% of human lifespan variation is explained by genetic factors [159], emphasizing the importance of the interactions between genetic background and environmental factors [221]. In animal models, some physiological and metabolic traits, especially lifespan, are strongly affected by genetic backgrounds and variations as well as non-genetic factors such as symbiotic microbiome and water balance [222]. When a collection of recombinant inbred mouse strains were tested for lifespan under ad libitum diet and CR (40% reduction compared to ad libitum diet) diet, a wide range of lifespan responses were observed in both ad libitum and CR diets [223,224]. For example, the mean lifespan of female mice on ad libitum diet varied from 407 to 1208 days. Strikingly, their lifespans on CR diet varied to a greater degree from 113 to 1225 days. Importantly, not only did CR fail in lifespan extension in some lines, but it even shortened lifespan in some lines too [223]. Similarly, a strong variation in lifespan response to diets was observed when a collection of nearly 200 genetically distinct lines of Drosophila (DGRP: Drosophila Genetic Reference Panel) tested for lifespan in ad libitum (5% Yeast) and CR (0.5% Yeast) [225]. In both cases, lifespan response also significantly varied between males and females [223,225], generating a further layer of complication in understanding the mechanisms of CR. A simple interpretation of these animal studies would suggest that a certain type of CR and IF may not be beneficial, but they can be even deleterious depending on genetic variations and sex [32]. Therefore, for human applications of CR and IF, we suggest that individualized genomics and medicine should be established first to take full advantage of CR and IF.
